# Melatonin Induces Melanogenesis in Human SK-MEL-1 Melanoma Cells Involving Glycogen Synthase Kinase-3 and Reactive Oxygen Species

**DOI:** 10.3390/ijms21144970

**Published:** 2020-07-14

**Authors:** Juan Perdomo, Carlos Quintana, Ignacio González, Inmaculada Hernández, Sara Rubio, Juan F. Loro, Russel J. Reiter, Francisco Estévez, José Quintana

**Affiliations:** 1 Departamento de Bioquímica y Biología Molecular, Instituto Universitario de Investigaciones Biomédicas y Sanitarias (IUIBS), Universidad de las Palmas de Gran Canaria, 35016 Las Palmas, Spain; juan.perdomo@ulpgc.es (J.P.); cqr1991@gmail.com (C.Q.); ignacio.gonzalez@ulpgc.es (I.G.); servanda.hernandez@ulpgc.es (I.H.); sara.rubio@ulpgc.es (S.R.); francisco.estevez@ulpgc.es (F.E.); 2 Departamento de Ciencias Clínicas, Universidad de las Palmas de Gran Canaria, 35016 Las Palmas, Spain; juanfrancisco.loro@ulpgc.es; 3 Department of Cellular and Structural Biology, The University of Texas Health Science at San Antonio, San Antonio, TX 78229, USA; reiter@uthscsa.edu

**Keywords:** melatonin, melanogenesis, melanoma, SK-MEL-1, GSK-3β, tyrosinase

## Abstract

Melatonin is present in all living organisms where it displays a diversity of physiological functions. Attenuation of melanogenesis by melatonin has been reported in some mammals and also in rodent melanoma cells. However, melatonin may also stimulate melanogenesis in human melanoma cells through mechanisms that have not yet been revealed. Using the human melanoma cells SK-MEL-1 as a model, an increase in both tyrosinase activity and melanin was already observed at 24 h after melatonin treatment with maximal levels of both being detected at 72 h. This effect was associated with the induction in the expression of the enzymes involved in the synthesis of melanin. In this scenario, glycogen synthase kinase-3β seems to play a significant function since melatonin decreased its phosphorylation and preincubation with specific inhibitors of this protein kinase (lithium or BIO) reduced the expression and activity of tyrosinase. Blocking of PI3K/AKT pathway stimulated melanogenesis and the effect was suppressed by the inhibitors of glycogen synthase kinase-3β. Although melatonin is a recognized antioxidant, we found that it stimulates reactive oxygen species generation in SK-MEL-1 cells. These chemical species seem to be an important signal in activating the melanogenic process since the antioxidants *N*-acetyl-l-cysteine and glutathione decreased both the level and activity of tyrosinase stimulated by melatonin. Our results support the view that regulation of melanogenesis involves a cross-talk between several signaling pathways.

## 1. Introduction

Melatonin is a physiological mediator in animals, bacteria, fungi, and plants [1]. In mammals, the pineal gland secretes melatonin into the blood and cerebrospinal fluid to exert a variety of well-documented physiological functions [2]. Among the vast array of biological functions exhibited by melatonin, its antineoplastic properties have attracted significant attention involving cell death, oncostatic properties, and differentiation [3,4,5].

In a previous report on human melanoma cells, we observed that melatonin reduces the number of cells and induces melanogenesis, a complex biosynthetic pathway involving enzymatic and non-enzymatic reactions to produce the skin pigment melanin [6]. In mammals, melanin is synthesized in the epidermal melanocytes and the pigment is then transferred to keratinocytes to allow skin and hair pigmentation [7]. In melanocytes, melanin biosynthesis is stimulated by α-melanocyte-stimulating hormone (α-MSH) and adrenocorticotropic hormone (ACTH) and non-hormonal effectors, including cAMP elevating agents, glycogen synthase kinase-3 (GSK-3) signaling pathway blocker, and by UV light. Melanin plays a photo-protective role against the carcinogenic effects of ultraviolet radiation, especially UVB [8]. The balance of a variety of signal transduction pathways regulates melanogenesis [9].

Pigment synthesis involves the conversion of tyrosine to melanin with the rate-limiting step, the oxidization of tyrosine to L-3,4-dihydroxyphenylalanine (L-DOPA), being catalyzed by tyrosinase. Two other key enzymes involved in the formation of melanin are tyrosinase-related protein 1 (TRP–1) and tyrosinase-related protein 2 (TRP-2) [10]. The pigmentation genes are transcriptionally regulated by microphthalmia-associated transcription factor (MITF), the main regulator of melanogenesis [11]. The *MITF* gene organization allows the generation of several protein isoforms differing at their N termini. Some isoforms can be found in many cell types while others exhibit a tissue-restricted pattern of expression. Such is the case of MITF-M, the specific and most abundant MITF isoform in melanocytes and melanoma cells [12]. The transcriptional activity of MITF also depends on its posttranslational modifications, mainly phosphorylation, and availability of co-operating factors. Phosphorylations of MITF by ERK1/2, p38, p90RSK, AKT and GSK-3β have been reported [13,14]. The expression levels of MITF is controlled by a range of transcription factors and their regulators associated with signaling pathways involved in a variety cellular processes [13]. Thus, cellular context and tumor microenvironment are key factors influencing MITF expression and activity [15]. A high number of genes encoding proteins with diverse functions have been identified as targets of MITF which can promote proliferation, cell survival, senescence, and differentiation-associated functions, including regulation of genes implicated in cell adhesion or pigmentation [16].

Phosphatidylinositol 3-kinase (PI3K)/AKT signaling is an important pathway for controlling melanogenesis and it is frequently found to be active in melanoma cells. In response to hormones and growth factors, the serine/threonine protein kinase AKT binds to PI3K phospholipid product phosphatidylinositol 3,4,5-trisphosphate on the plasma membrane where it is activated by phosphorylation on threonine 308 and serine 473 [17]. The properties of a wide range of proteins are sensitive to phosphorylation by AKT. A recognized target is GSK-3β, a protein serine/threonine kinase involved in cell signaling which is phosphorylated on serine 9 leading to its inactivation [18]. Herein, we demonstrate that melatonin upregulates the enzymes involved in melanogenesis in SK-MEL-1 and provide evidences that GSK-3β plays a central role. Moreover, although melatonin is known as a radical scavenger, reactive oxygen species seems to be involved in the melanogenic process since they are quickly stimulated by the indoleamine and melanogenesis is blocked by the use of antioxidants.

## 2. Results

### 2.1. Melatonin Stimulates the Expression of Tyrosinase and Tyrosinase-Related Protein-1

**Figure 1:**
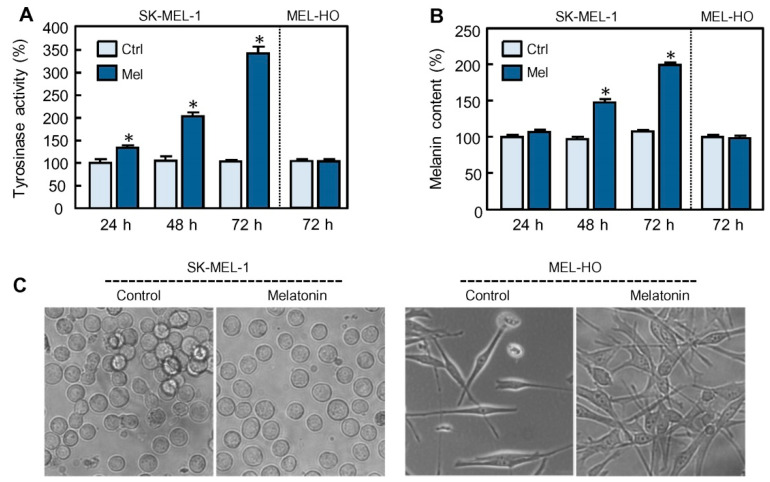
Effect of melatonin on melanogenesis in human melanoma cells SK-MEL-1 and MEL-HO. The cells were cultured in presence of 1 mM melatonin (Mel) for the time period indicated and tyrosinase activity (**A**) and melanin content (**B**) were determined and expressed as percentage of control. (**C**) The cells were cultured in presence of 1 mM melatonin for 72 h and visualized under phase contrast microscopy; original magnification 20×. * *p* < 0.05 vs. control (Ctrl).

In a previous report we showed that melatonin, used at a high concentration compared to the levels in blood [19], induces melanogenesis through a non-receptor mediated mechanism in SK-MEL-1 cells, a human melanoma cell line with capacity to produce melanin [6]. The aim of the present study was to identify signaling pathways involved in the stimulation of melanogenesis by the indoleamine. To this end, we first evaluated the kinetics of induction of melanogenesis in response to melatonin. As shown in Figure 1A melatonin (1 mM) stimulated tyrosinase activity, the enzyme which catalyzes the limiting step of melanogenesis, in a time-dependent manner. A slight increase in tyrosinase activity (1.3-fold) was already detected at 24 h of incubation with the indoleamine and maximal levels (~3.5-fold) were achieved with the longest incubation time (72 h). In agreement with this observation, melanin content also augmented in melatonin-treated cells as compared to control cells; a clear increase (1.5-fold) was already detected at 48 h and a higher rise (2–fold) at 72 h (Figure 1B). The periods of times required to detect these biochemical changes in response to melatonin in SK-MEL-1 cells were similar to the reported for a variety of stimulators in different human and murine melanoma cell lines [20]. In contrast, changes in tyrosinase activity and melanin synthesis were not detected in MEL-HO (Figure 1A,B), a human epithelial-like adherent melanoma cell line that, however, exhibits increased dendricity in response to the indoleamine (Figure 1C). A mutation in BRAF gene is found in approximately 50% of melanoma, resulting in a constitutive activation of RAF-MEK-MAPK pathway, leading to cellular proliferation, survival and differentiation [21]. SK-MEL-1 and MEL-HO cells have been reported to contain the BRAF V600E mutation [22,23]. Our studies revealed that these cells are highly sensitive to melatonin-induced cell growth inhibition since the number of cells was reduced to ~50% following 72 h of treatment. Melatonin was not cytotoxic against these cells, so the reduction in cell number is associated with cell growth arrest and/or differentiation. Because SK-MEL-1 cells exposed to melatonin acquired an elevated melanogenic capacity at 72 h, most studies to determine the underlying mechanism involved were evaluated at this time.

**Figure 2:**
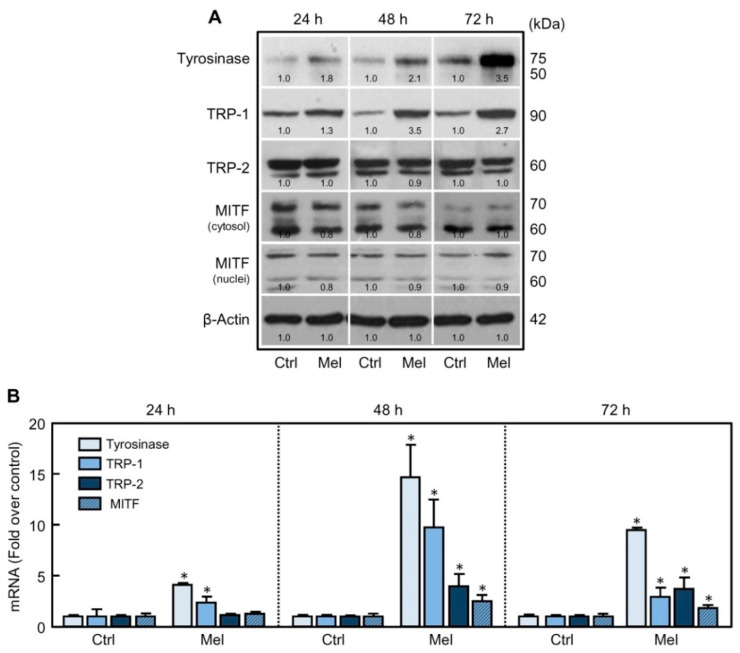
Effect of melatonin on the expression of melanogenesis-related proteins and pigmentation-related genes. (**A**) The cells were incubated with 1 mM melatonin (Mel) for the period of time specified and tyrosinase, TRP-1, TRP-2, and MITF were analyzed by immunoblotting; as a loading control, β-actin was also determined. (**B**) The cells were treated as in (A) and the mRNA levels of tyrosinase, TRP-1, TRP-2, and MITF were determined by real-time quantitative RT-PCR analysis; data were normalized to endogenous glyceraldehyde 3-phosphate dehydrogenase expression levels and expressed as fold changes over control group. * *p* < 0.05 vs. control group (Ctrl).

Tyrosinase is an enzyme subjected to posttranslational modifications; hence, tyrosinase activity rather than total tyrosinase protein is usually correlated with melanin production [24]. To determine whether the increase in tyrosinase activity in response to melatonin is also associated with changes in the levels of the enzymes involved in melanogenesis, lysates from SK-MEL-1 cells were analyzed by immunoblotting. An increase in tyrosinase, and also in TRP-1, was already detected at 24 h and it remained elevated for at least 72 h (Figure 2A); in contrast, changes in the levels of TRP-2 were not detected at any time analyzed. Next, we performed real-time quantitative RT-PCR to determine whether the augmentation of melanogenic proteins was due to an increased mRNA expression. As shown in Figure 2B, the mRNA levels of tyrosinase and TRP-1 increased at 24 h in the cells exposed to melatonin as compared to untreated cells, and the maximum induction (15-fold for tyrosinase and 10-fold for TRP-1) was observed after 48 h of treatment. In accordance with the results shown in Figure 2A, the levels of transcripts for TRP-2 were slightly modified by melatonin.

The MITF transcription factor is considered the main transcriptional regulator of melanogenesis and it has been shown to play a key role in melanocyte differentiation through the direct transcriptional control of *tyrosinase*, *TRP-1* and *TRP-2* genes [11]. To determine whether melatonin stimulates the expression of MITF protein, the cells were treated with the indoleamine for 24–72 h and cytosolic and nuclear fractions analyzed by immunoblotting. A doublet of about 60 kDa and a 70 kDa bands was detected in control cells (Figure 2A), which represents multiple MITF isoforms that are known to be expressed from distinct promoters of the *MITF* gene [25]. The expression of this transcription factor, however, appears not to be affected by melatonin at the time-frame analyzed (Figure 2A) even though a slight increase in the mRNA levels by the indoleamine was detected at 48 h and 72 h (Figure 2B). The reason of these differences remains to be elucidated.

### 2.2. Glycogen Synthase Kinase-3 Inhibition Blocks Melanogenesis Stimulated by Melatonin

**Figure 3:**
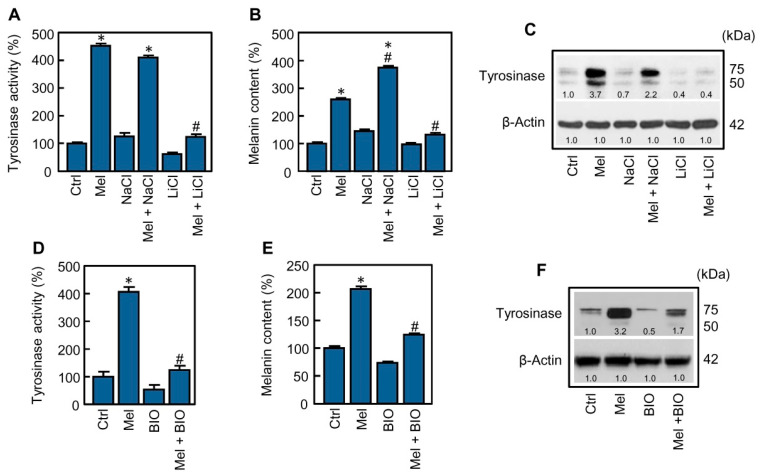
Effect of GSK-3 inhibitors on activation of melanogenesis by melatonin. (**A**–**C**) The cells were pre-incubated with 20 mM LiCl or 20 mM NaCl for 2 h and then treated with 1 mM melatonin (Mel) for 72 h. Tyrosinase activity (A) and melanin content (B) were determined and the values expressed as percentages of control. Tyrosinase levels (C) were analyzed by immunoblotting and β-actin was included as a loading control. (**D**–**F**) The cells were pre-incubated in absence or presence of 0.5 µM BIO for 2 h and then treated with or without 1 mM melatonin (Mel) for 72 h. Tyrosinase activity (D) and melanin content (E) were measured and the values expressed as percentages of control. The expression of tyrosinase (F) was determined by immunoblotting from lysates. * *p* < 0.05 vs. control (Ctrl); # *p* < 0.05 vs. melatonin treatment alone.

MITF is a recognized target of a wide range of protein kinases involved in cell signaling and it is accepted that its phosphorylation may enhance its transcriptional activity [12]. Glycogen synthase kinase-3β is a multi-functional kinase in many signaling pathways that has been involved in the regulation of melanogenesis, in human and rodent cellular models [26]. In the next experiment, the role of GSK-3β in the stimulation of melanogenesis by melatonin was explored by using two widely recognized pharmacological inhibitors, lithium and the hemi-synthetic and cell-permeable indirubin derivative BIO. The classic glycogen synthase kinase-3 inhibitor lithium (LiCl, 20 mM) completely blocked the stimulatory effect of melatonin on tyrosinase activity (Figure 3A) and on melanin synthesis (Figure 3B); in accordance with these results, lithium also reduced melatonin-induced expression of tyrosinase to basal levels as revealed by the immunoblotting analysis (Figure 3C). In this study, sodium chloride was also included as a control. Similar results were observed in presence of BIO (0.5 µM), considered as a more specific inhibitor of GSK-3. This inhibitor abrogated both tyrosinase activity (Figure 3D) and melanin synthesis (Figure 3E) induced by melatonin; thus, as observed with lithium, the immunoblotting analysis indicated that the levels of tyrosinase detected after stimulation with the indoleamine were also reduced to the basal level in presence of BIO (Figure 3F). Neither lithium nor BIO were cytotoxic for SK-MEL-1 at the concentration used in the present study. Together, these results suggest an important role of GSK-3 in the activation of melanogenesis by melatonin.

**Figure 4:**
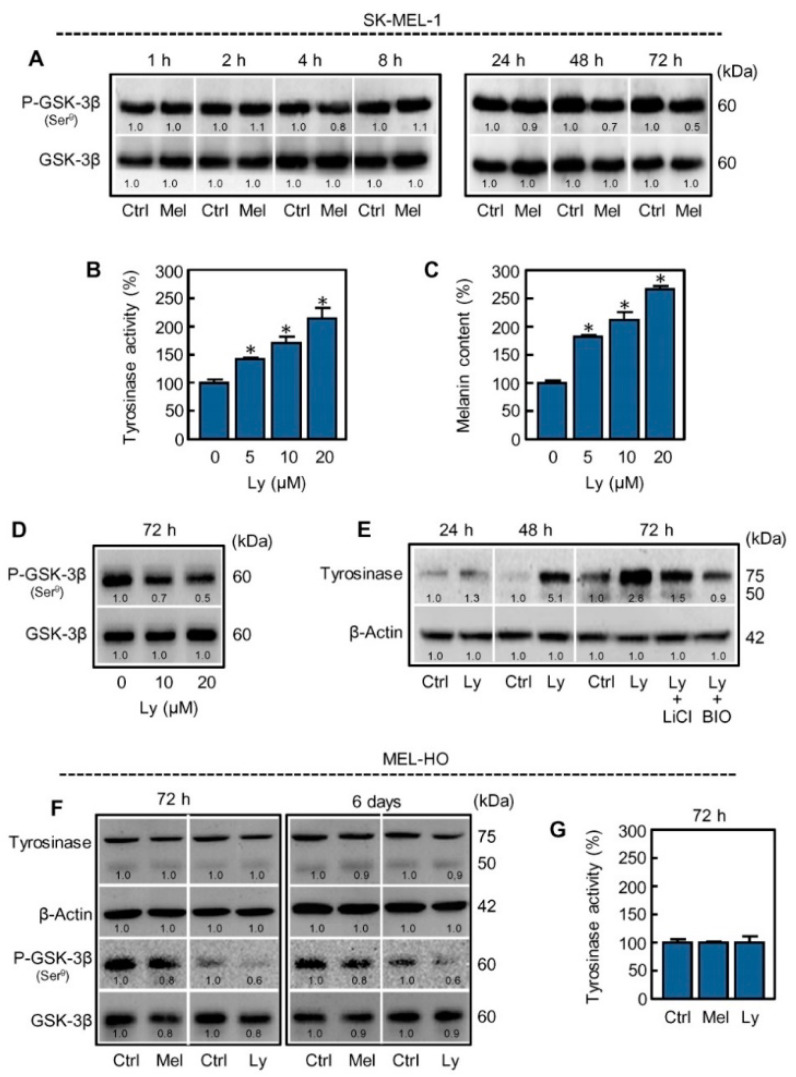
Melatonin reduces the levels of P-GSK-3β and Ly294002 mimics the effect of melatonin on melanogenesis in SK-MEL-1 cells. (**A**) The cells were incubated in presence of 1 mM melatonin (Mel) for the indicated period of time and P-GSK-3β was analyzed by immunoblotting. Membranes were stripped and reprobed with a total GSK-3β antibody as a loading control. (**B,C**) The cells were incubated with the specified concentrations of the PI3K inhibitor Ly294002 (Ly) for 72 h and tyrosinase activity and melanin content evaluated using a microplate reader. (**D**) The cells were treated with the indicated concentrations of Ly294002 (Ly) and P-GSK-3β was analyzed by immunoblotting. (**E**) The cells were pre-incubated in presence of 20 mM LiCl, or 0.5 µM BIO, for 2 h and then they were treated with 20 µM Ly294002 for the indicated period of time. Expression of tyrosinase was evaluated by immunoblotting and the level of β-actin was also measured as a loading control. (**F**) HO-MEL cells were incubated with 1 mM melatonin or 20 µM Ly294002 (Ly) for the indicated period of time and lysates were analyzed by immunoblotting. (**G**) The cells were treated as above and tyrosinase activity was determined using a microplate reader. * *p* < 0.05 vs. untreated cells.

In mammals GSK-3 is expressed as two isozymes, GSK-3α and GSK-3β; however, most biochemical studies have focused on GSK-3β because knockout mice of this isoform are embryonically lethal. The activity of GSK-3β is regulated by phosphorylation on serine-9, which is inhibited because the phosphorylated N-terminal tail acts as a pseudosubstrate sequence [27]. To determine whether melatonin has an impact on phosphorylation of GSK-3β, cells were treated with the indoleamine for short (1–8 h) or long (24–72 h) time periods and cell lysates analyzed by immunoblotting. In agreement with the above results, melatonin markedly decreased the levels of phosphorylated GSK-3β at 48 h and 72 h, as compared to the respective untreated controls, whereas total GSK-3β remained unchanged along the time-course (Figure 4A). These results suggest that dephosphorylation and thereby activation of GSK-3β seems to play a significant role in melanogenesis triggered by melatonin.

### 2.3. Inhibition of PI3K/AKT Pathway Mimics the Effect of Melatonin on Melanogenesis in SK-MEL-1 Cells

GSK-3β can be phosphorylated by a variety of kinases including AKT, PKA, PKC, and p70 S6 kinase [27]. Therefore, multiple signaling pathways converge on GSK-3β which acts as an integrator of signals. Since the PI3K/AKT pathway is frequently activated in melanoma cells, we evaluated whether its blockade reproduces the effect of melatonin. To this end, cells were incubated with the PI3K specific inhibitor Ly294002 for 72 h and tyrosinase activity, intracellular melanin amount, and phospho-GSK-3β were analyzed from lysates. The results indicate that Ly294002 induces tyrosinase activity (Figure 4B), increases melanin content (Figure 4C) and decreases the levels of phospho-GSK-3β (P-GSK-3β) in a concentration dependent manner (Figure 4D). A similar trend in the expression of tyrosinase was observed in the cells treated with Ly294002 (Figure 4E) as compared with the cells incubated with melatonin (Figure 2A). Consistent with the hypothesized mechanism, the stimulatory effect of Ly294002 on tyrosinase expression was also blocked by the GSK-3 inhibitors lithium and BIO (Figure 4E). These results strongly suggest that the PI3K/AKT pathway is involved in the activation of GSK-3β by melatonin in SK-MEL-1.

To determine whether the effect of melatonin on GSK-3β activation in SK-MEL-1 is shared with another melanoma cell line, we have included MEL-HO in the present study. Melatonin and Ly294002 also induced a reduction in P-GSK-3β in MEL-HO, which was accompanied with a slight decrease in the expression of GSK-3β; however, there were no obvious changes in the activity or expression of tyrosinase (Figure 4F,G). Melanogenic inducers like α-MSH of But_2_-cAMP were not able to induce melanogenesis in MEL-HO cells either. The fact that melanogenesis is not stimulated in these cells may be due to additional mutations in genes and/or downregulation of factors involved in the complex melanogenic process. However, morphological changes that are frequently associated with melanogenesis activation were observed by phase contrast microscopy in MEL-HO cells as shown above (Figure 1C). In this context, it is interesting to note that we observed a band corresponding to the non-functional (unglycosylated) 50 kDa-form of tyrosinase, a low expression of the 75 kDa-mature form of tyrosinase and also a low tyrosinase activity in MEL-HO as compared with SK-MEL-1. Together, the results suggest that melatonin modulates the PI3K/AKT pathway and activates GSK-3β in both SK-MEL-1 and MEL-HO melanoma cells.

### 2.4. Reactive Oxygen Species Are Involved in the Stimulatory Effect of Melatonin on Melanogenesis

**Figure 5:**
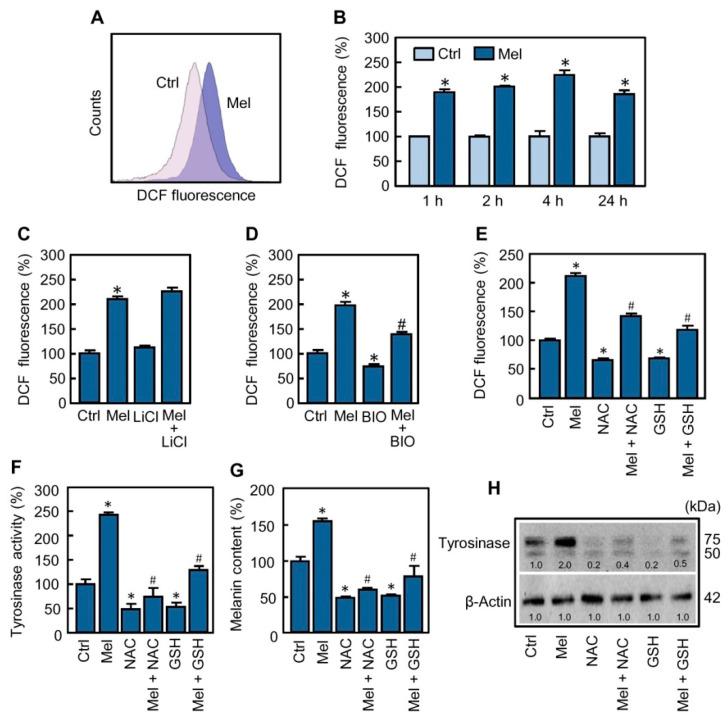
Reactive oxygen species are involved in melanogenesis stimulated by melatonin. (**A**) Cells were cultured with 1 mM melatonin (Mel) for 2 h, stained with H_2_-DCF and the fluorescence emitted by the oxidized fluorochrome was determined by flow cytometry; a representative histogram is shown. (**B**) Cells were treated with 1 mM melatonin for the time interval indicated and analyzed as above; mean fluorescence intensity from the resulting histograms was determined and the values are expressed as percentages of control. (**C**,**D**) Cells were pre-incubated with 20 mM LiCl, or 0.5 µM BIO, for 2 h and then they were treated with 1 mM melatonin for 4 h; DCF-derived fluorescence was determined as in (B). (**E**) The cells were pre-incubated with 5 mM NAC or GSH for 2 h and then treated with 1 mM melatonin for 4 h; relative ROS levels were determined by flow cytometry. (**F**–**H**) The cells were pre-treated with 5 mM NAC or GSH for 2 h and then treated with 1 mM melatonin for 72 h. Tyrosinase activity (F) and melanin content (G) were measured from lysates in a microplate reader. (H) Expression of tyrosinase was analyzed by immunoblotting and β-actin was included as a loading control. * *p* < 0.05 vs. the respective control; # *p* < 0.05 vs. melatonin treatment alone.

Although most studies indicate that melatonin exhibits antioxidant properties, there are increasing evidences in cancer cells that the indoleamine displays pro-oxidant activity [28] and that the reactive oxygen species (ROS) could play a role in the regulation of melanogenesis in melanoma cells [20,29,30]. To determine whether melatonin stimulates reactive oxygen species production in SK-MEL-1, treated cells were incubated for different time periods and intracellular levels of these chemical species were analyzed by flow cytometry using the H_2_-DCF fluorescent probe. As shown, melatonin induced generation of ROS (Figure 5A) and the maximal production (~2.3-fold increase compared with untreated cells) was reached at 4 h of treatment (Figure 5B). The formation of ROS was an early event since it was detected 1 h after treatment and the levels of these reactants remained elevated for at least 24 h. These chemical species have been involved as mediators in the cytotoxicity of melatonin, alone or in combination with known antitumoral compounds, against a variety of tumor cells [31,32,33]. Cell viability of SK-MEL-1 was not affected by melatonin treatment for the 24–72 h period, which suggests that ROS generated in response to the indoleamine do not modulate cell death in this melanoma cell model.

**Figure 6:**
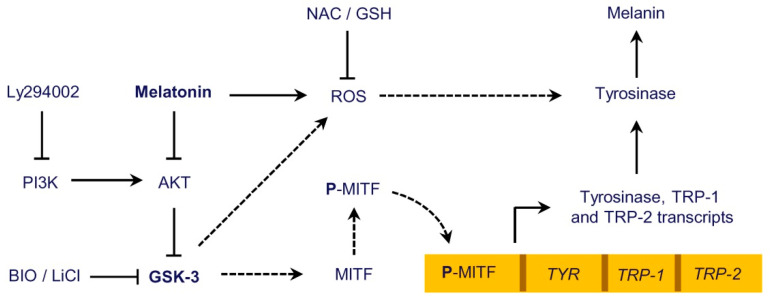
Schematic summary of the present study. Melatonin via PI3K/AKT pathway inhibition promotes GSK-3β dephosphorylation (i.e., activation) which probably phosphorylates the transcription factor MITF at serine 298, enhancing its transcriptional activity. MITF binds to *tyrosinase*, *TRP-1* and *TRP-2* promoters, leading to the increase in the expression of respective transcripts. Specific inhibitors of GSK-3β, like BIO and LiCl, block the effect of melatonin on the expression and activity of tyrosinase and on the synthesis of melanin. Melatonin also induces ROS generation that is involved in tyrosinase activity stimulation and melanin synthesis; the glycogen synthase kinase-3β inhibitor BIO and ROS scavengers like *N*-acetyl-L-cysteine (NAC) and glutathione (GSH) reduce ROS formation and melanogenesis induction. The PI3K specific inhibitor Ly294002 reproduces the effect of melatonin on GSK-3β activation and on tyrosinase activity and melanin production.

It is known that the levels of ROS are regulated by ROS-metabolizing enzymes. In particular, nuclear factor erythroid 2-related factor 2 (Nrf2) is a transcription factor that influences the expression of several enzymes involved in the elimination of ROS and, in addition to redox interactions with Kelch like ECH associated protein 1 (Keap1), can be regulated either directly or indirectly by GSK-3β [34,35]. Since GSK-3β seems to be a mediator in melanogenesis in SK-MEL-1, we investigated whether this protein kinase has an influence on the ROS formation triggered by melatonin. For this purpose, the cells were pre-incubated with the GSK-3β inhibitors lithium (LiCl, 20 mM) or BIO (0.5 µM) for 2 h and then treated with the indoleamine for 4 h. The results (Figure 5C,D) indicate that BIO, but not lithium, blocked to a large extent the generation of ROS promoted by melatonin and reduced the basal levels of these chemical species. Because GSK-3β modulates a large variety of signaling pathways, to further clarify the role of ROS in the activation of melanogenesis, the cells were pre-incubated with *N*-acetyl-L-cysteine (NAC, 5 mM) or glutathione (GSH, 5 mM) for 2 h and then treated with 1 mM melatonin for 4 h (for determining ROS) or 72 h (for determining activity and expression of tyrosinase and melanin content). The results support an important function of ROS in triggering melanogenesis since the above antioxidants decreased melatonin-induced intracellular ROS formation (Figure 5E), tyrosinase activity (Figure 5F) and melanin content (Figure 5G). Moreover, the effect of NAC and GSH on melanogenesis activated by melatonin involves a reduction in the levels of mature tyrosinase protein as revealed by the immunoblotting studies (Figure 5H). It seems that ROS generation is also an important signal to maintain the basal levels of melanogenic activity observed in SK-MEL-1 because the treatment with NAC or GSH alone provoked a clear reduction of ROS which was accompanied with a decrease in tyrosinase activity, intracellular melanin content and also in the expression of tyrosinase protein (Figure 5E–H). Together, these results suggest a central role of GSK-3β in melatonin-induced and ROS-mediated melanogenesis in SK-MEL-1 (Figure 6).

## 3. Discussion

Melatonin displays a variety of biological properties and one of the first effects described was the lightening of the skin of amphibians. Melatonin also attenuates melanogenesis in some mammals and it stimulates or reduces melanogenesis in cultured cells from rodent melanomas [36,37]. In human melanocytes, melatonin appears not to have a major influence on the pigmentation [38]. However, in human melanoma cells melatonin may both stimulate tyrosinase activity and pigmentation, as described in SK-MEL-28 and SK-MEL-1 cells [6,39], or decrease melanogenesis as reported in MNT-1 melanoma cells [3]. The basis of differential response of melanoma cells to melatonin treatment is unknown and it could be multifactorial. For example, melatonin may be metabolized through indole and kynurenic pathways to N^1^-acetyl-N^2^-formyl-5-methoxykinuramine, 6-hydroxymelatonin and 5-methoxytryptamine in melanoma cells [40], and that the latter metabolite stimulates tyrosinase activity and intracellular melanin content in human SK-MEL-188 [38]. Therefore, in addition to cellular context differences, the identification of intracellular signaling pathways activated in response to melatonin will provide knowledge about their influence on melanogenic process on melanoma cells. In the present study, we investigated the mechanism used by melatonin to stimulate melanogenesis on SK-MEL-1. The results demonstrate that melatonin-stimulated melanogenesis involves tyrosinase and TRP-1 upregulation, which was associated with an increase in the mRNA. Thus, maximum levels of the enzymes were observed at 72 h of treatment while the transcripts peaked earlier, at 48 h; this probably reflects translational regulation and/or the posttranslational modifications (i.e., glycosylation) required to get mature enzymes [41]. It should be noted that only tyrosinase is absolutely necessary for melanogenesis and differential regulation may explain the lack of effect of melatonin on TRP-2 expression.

The stimulation of the biosynthesis of the melanogenic enzymes usually involves upregulation of MITF which is controlled by a variety of transcription factors and their associated regulators [13]. The immunoblotting studies on SK-MEL-1 revealed that MITF expression is not modulated by melatonin since no changes were detected in the cytosol or nuclei at any time analyzed. It is known that the transcriptional activity of MITF also depends on posttranslational modifications and availability of cooperating protein factors [12]. Therefore, we investigated the role of GSK-3β since it increases the transcriptional activity of MITF by phosphorylation at serine 298 [26]. The exchange of this amino acid residue for a proline due to a mutation in the *MITF* gene is the cause of the Waardenburg syndrome type II and reveals the central role displayed by GSK-3β in the regulation of pigmentation [42]. Two pharmacological inhibitors with different inhibitory mechanisms were used to block the activity of GSK-3β, lithium, and BIO. Lithium displays affinity for the Mg^+2^-binding site and it has been long used in the treatment of bipolar disorders, and the indirubin analog, BIO, exhibits affinity for the ATP-binding domain [43,44]. Both inhibitors were equally effective in the inhibition of melanogenesis. This strongly suggests that melatonin may stimulate the transcriptional activity of MITF by modulating the GSK-3β activity. This enzyme is an important member of the β-catenin destruction complex that phosphorylates β-catenin as a preceding step for its degradation, keeping the cytosolic concentrations of β-catenin low in the absence of Wnt ligands. It has been reported that β-catenin may collaborate to stimulate MITF transcription and therefore promote melanogenesis [13,45]. Consistent with the results on the expression of MITF (Figure 2A) changes in the levels of β-catenin were not observed upon stimulation with melatonin in our cell model. Thus, the treatment of the cells with lithium or BIO alone, that would be expected to increase β-catenin due to GSK-3β inhibition, did not stimulate the expression of tyrosinase. In contrast, both inhibitors reduced the basal levels of tyrosinase (Figure 3C,F).

The PI3K/AKT pathway is frequently activated in the human melanoma cells and GSK-3β is inhibited as a result of phosphorylation of serine 9 by AKT [46]. The importance of this pathway to promote melanogenesis was established by Khaled et al. in B16-F10 murine melanoma cells treated with Ly294002, a PI3K specific inhibitor widely used to delineate the physiological functions of this route [47]. We found that melatonin promotes a reduction in the phosphorylation of serine 9 (i.e., activation) of the enzyme at 48 h and 72 h after treatment. In accordance with the role of PI3K/AKT pathway, incubation of SK-MEL-1 with Ly294002 decreased the phosphorylation of GSK-3β and reproduced the effect of melatonin on melanogenesis. In this context, it has been reported that melatonin reduces the levels of phospho-AKT in tumor cells [33,48]. Together, these results suggest that melatonin modulates the activity of GSK-3β to stimulate pigmentation through the PI3K/AKT pathway. It has been also reported that MITF is phosphorylated by AKT at serine 510 in melanoma cells, leading to an unstable transcription factor and decreased tyrosinase expression [14]. Although we cannot rule out an impact of melatonin on dephosphorylation of MITF at serine 510 that may lead to an increase in tyrosine activity, the results exposed herein are more consistent with a central role of the AKT downstream target GSK-3β. Moreover, since GSK-3β may also be phosphorylated on serine 9 by other kinases such as PKA and PKC, among others [49], future studies will be required to determine their role in the activation of melanogenic process.

Melatonin is a powerful antioxidant itself that also stimulates the anti-oxidant defenses in normal cells, playing therefore a protective role [28,50]. However, in tumor cells melatonin often exhibits pro-oxidant properties which is supported by a large number of studies on leukemia cells, cervical cancer cells, hepatocellular carcinoma cells, melanoma cells, and thyroid cells, among others [31,32,51]. Thus, most investigations indicate that the cytotoxic effects of melatonin are attributed to its ability to stimulate free radical generation which causes cell death. Our results demonstrate that melatonin promotes acute and persistent formation of ROS and that these chemical species are important mediators in the activation of melanogenesis. Reactive oxygen species are involved in the activation of melanogenesis in melanocytes exposed to UVB [52] and this pathway is also stimulated in murine B16F10 and human SK-Mel-2 melanoma cells exposed to hydrogen peroxide through a mechanism involving upregulation of ATP synthase β [53,54]. Because melatonin is nontoxic to SK-MEL-1 cells, the cellular context appears to be important for the ROS generated by the indoleamine treatment. Thus, SK-MEL-1 cells express basal levels of MITF (Figure 2A), and it is generally accepted that melanoma cells with high amounts of this transcription factor are less vulnerable to ROS [55].

In the present study, GSK-3β was also involved in the increase of ROS, since its inhibition (at least with BIO) partially abrogated the generation of ROS in response to melatonin. It is known that GSK-3β may play a critical role in regulating Nrf2, the master regulator of endogenous antioxidant responses [56]. Under non-stressful conditions Nrf2 binds to Keap1 in the cytosol where is continuously degraded by the proteasome. During stress Keap1 is oxidized and Nrf2 translocates to the nucleus where it induces the transcription of target genes. Several reports indicate that GSK-3β may stimulate the degradation of Nrf2, thereby promoting an elevation of ROS [34,35]. Interestingly, it has been reported that melatonin and its derivatives protect melanocytes against UVB-mediated ROS injury via Nrf2 activation [57,58]. It is possible that the effect of melatonin in normal cells involves activation of the PI3K/AKT pathway and therefore inactivation of GSK-3β [59,60] in contrast to the observed effect in the SK-MEL-1 and other cancer cells [48,61].

The involvement of GSK-3 in the elevation of ROS by melatonin needs to be clarified since increases in ROS levels apparently preceded the changes in the phosphorylation state of GSK-3β, although it has been speculated that phospho-GSK-3 may be transiently activated whether the levels of a particular target increase enough to efficiently compete with the inhibitory N-terminal peptide [27]. Also, although BIO and lithium efficiently blocked melanogenesis, only BIO reduced ROS. It should be noted that BIO and lithium inhibit both isoform of GSK-3; however, the functions of GSK-3α and GSK-3β are not redundant and there are differences in signaling mechanisms that regulate the two isoforms [62].

In summary, herein we report that melatonin stimulates ROS generation and GSK-3β activity which in turn promote melanogenesis in SKMEL-1 cells. Activation of melanogenesis by melatonin in melanoma cells is likely more frequent than described so far; for example, Alvarez-Artime et al. reported recently that the indoleamine stimulates pigmentation in murine BF16-F10 melanoma cells [36]. However, we have also explored the effect of melatonin on MEL-HO—a human melanoma cell line that is resistant to melanogenic stimuli—and found that the indoleamine reduced the cell proliferation without evidences of toxicity or melanogenesis induction. Our results on SK-MEL-1 could stimulate interest in the role of melanogenesis activation by melatonin and the impact on cell growth inhibition on additional melanoma cell lines. In this context, an interesting question to answer is whether the SK-MEL-1 cells exposed to melatonin are more sensitive to cytotoxic properties of conventional antitumor agents since overexpression of tyrosinase has been reported to sensitize human melanoma cells to dacarbazine, an alkylating agent that is used in the therapy against metastatic malignant melanoma usually in combination with other antineoplastic agents [63]. Since melatonin is a tyrosinase activity inducer, it could be of clinical interest to determine whether the indoleamine modulates the sensitivity of human melanoma cells to conventional chemotherapy. This is an important issue because melatonin has proven to be safe during clinical use when administered as a coadjuvant in chemotherapy and also in radiotherapy [64]. Despite the large number of studies demonstrating the formation of ROS induced by melatonin, the mechanism by which melatonin increases ROS levels in cancer cells remains unknown. Further studies are necessary to determine the mechanism of ROS formation and also whether melatonin derived metabolites are also involved in the activation of melanogenesis, such as described for 5-methoxytryptamine in human melanoma cells.

## 4. Materials and Methods

### 4.1. Reagents

Melatonin, L-DOPA, Ly294002, 2′,7′-dichlorodihydrofluorescein diacetate (H_2_-DCF), lithium chloride, and protease inhibitors were from Sigma (St Louis, MO, USA). The inhibitor (2’Z,3’E)-6-bromoindirubin-3’-oxime (BIO) was obtained from Tocris (Bristol, UK). Poly(vinylidene difluoride) (PVDF) membranes were purchased from Millipore (Billerica, MA, USA). Acrylamide, bisacrylamide, and the Bradford reagent were from Bio-Rad (Hercules, CA, USA). The following primary antibodies were from Santa Cruz Biotechnology (Dallas, TX, USA): tyrosinase mouse monoclonal antibody (cat no. sc-20035, RRID:AB_628420), TRP-1 rabbit polyclonal antibody (cat no. sc-25543, RRID:AB_2211150), TRP-2 rabbit polyclonal antibody (cat no. sc-25544, RRID:AB_793584), MITF mouse monoclonal antibody (cat no. sc-52938, RRID:AB_784546). Others primary antibodies were β-catenin mouse monoclonal antibody (Enzo Life Sciences, Farmingdale, NY, USA, cat no. ADI-KAM-ST001, RRID:AB_10615502), GSK-3β rabbit monoclonal antibody (Cell Signaling Technology, Danvers, MA, USA, cat no. 9315, RRID:AB_490890), phospho-GSK-3β (Ser9) rabbit monoclonal antibody (Cell Signaling Technology, cat no. 9322, RRID:AB_2115196). Secondary antibodies were obtained from GE Healthcare (Little Chalfont, UK).

### 4.2. Cell Culture Conditions

Human SK-MEL-1 (cat no. ACC-303, RRID:CVCL_0068) and MEL-HO (cat no. ACC-62, RRID:CVCL_1402) melanoma cell lines was purchased from DSMZ (Braunschweig, Germany). SK-MEL-1 are mostly round cells growing singly or in clumps in suspension while MEL-HO are epithelial-like adherent cells with long and narrow cell bodies. The cells were cultured in RPMI 1640 supplemented with 10% (*v*/*v*) fetal bovine serum, 100 U/mL penicillin and 100 μg/mL streptomycin, and maintained at 37 °C in a humidified atmosphere with 5% CO_2_. In all experimental procedures, cells were seeded at 2 × 10^5^ cells/mL and treated with 1 mM melatonin freshly dissolved in ethanol. The final concentration of vehicle, 0.5% (*v*/*v*), was non-toxic to the cells [6].

### 4.3. Tyrosinase Activity

Cells (~1–2 × 10^6^) were lysed on ice with 200 μL of PBS (pH 7.4) containing 1% Triton X-100 and lysates were centrifuged at 22,000× *g* for 10 min at 4 °C. Aliquots of supernatant (50 μL) were mixed with 50 μL of 2 mM L-DOPA, dissolved in 50 mM PBS (pH 6.8), and incubated at room temperature for 60 min. Tyrosinase activity was obtained by measuring the optical density at 475 nm in a microplate reader and normalized by the amount of protein.

### 4.4. Melanin Content

Cells (~1 × 10^6^) were solubilized with 250 μL of 1 M NaOH for 3 h at 60 °C and lysates were centrifuged at 22,000× *g* for 10 min. Melanin content of supernatants was determined by measuring the optical density at 405 nm and adjusted by the amount of protein.

### 4.5. Immunoblotting

To obtain whole cell lysates, cells were incubated on ice in lysis buffer (150 mM NaCl, 1% Triton X-100, 10% glycerol, 2 mM EDTA, and 20 mM Tris-HCl (pH 7.4)) containing protease and phosphatase inhibitors (1 mM PMSF, 5 μg/mL of leupeptin, aprotinin and pepstatin A, 10 mM sodium fluoride, 2 mM sodium orthovanadate, 2 mM tetrasodium pyrophosphate, 20 mM sodium β-glycerophosphate). The lysates were sonicated on ice and cleared by centrifugation at 22,000× *g* for 15 min at 4 °C. For the subcellular fractions preparations, cells were incubated on ice in isotonic buffer (250 mM sucrose, 10 mM KCl, 1.5 mM MgCl_2_, 1 mM EDTA, 1 mM EGTA, 1 mM DTT, 20 mM HEPES (pH 7.4)) containing protease inhibitors and lysed with a 22-gauge needle. Lysates were centrifuged at 1000× *g* for 5 min at 4 °C to obtain the nuclei and the resulting supernatants were centrifuged at 22,000× *g* for 15 min at 4 °C to obtain the cytosolic fraction. Protein content of each lysate was determined by the Bradford method. Samples containing equal amounts of proteins were resolved by 10% SDS-PAGE, electrotransferred to PVDF membranes and then incubated with specific antibodies at 4 °C overnight. Blots were incubated with the appropriate horseradish peroxidase-conjugated secondary antibody and proteins detected by chemiluminescence (Millipore, Billerica, MA, USA). The immunoblots were analyzed with ImageJ software (v.1.52u, NIH, Bethesda, MD, USA) to quantify the relative expression of each protein and data were normalized to the specified loading control.

### 4.6. Real-Time Quantitative RT-PCR

Total RNA was isolated using TRzol reagent (Life Technologies, Carlsbad, CA, USA) and reverse-transcribed with random hexamers (iScript™ cDNA Synthesis Kit, Bio-Rad). Real-time quantitative RT-PCR was performed with an iQ5 detector (Bio-Rad) using a dissociation protocol to evaluate the specificity of the primers and the consistency of the PCR products. Amplifications were performed in triplicates. The level of individual mRNA was normalized to the level of glyceraldehyde-3-phosphate dehydrogenase (GAPDH) expression. Forward and reverse primers were as follows: tyrosinase, 5′-AGTGTAGCCTTCTTCCAACTCAG-3′ and 5′-TTCCTCATTACCAAATAGCATCC; TRP-1, 5′-AAGGCTACAACAAAAATCACCAT-3′ and 5′-ATTGAGAGGCAGGGAAACAC-3′; TRP-2, 5′-GCAGCAAGAGATACACAGAAGAA and 5′-TCCTTTATTGTCAGCGTCAGA-3′; MITF, 5’-AAACCCCACCAAGTACCACA-3′ and 5′-ACATGGCAAGCTCAGGAC-3′; GADPH, 5′-GAGTCCACTGGCGTCTTCA-3′ and 5′- TTCAGCTCAGGGATGACCTT-3′.

### 4.7. Intracellular ROS Determination

After treatment, the cells were further incubated with 5 μM of fluorescent probe 2′,7′-dichlorodihydrofluorescein diacetate (H_2_-DCF) for 30 min, washed with ice-cold PBS and resuspended in the same buffer. Cells were analyzed on a BD FACSVerse™ Flow Cytometer (Becton-Dickinson, San José, CA, USA).

### 4.8. Statistical Analysis

All determinations were carried out in triplicate, and the data shown are representative results from at least three independent experiments. Data were analyzed with Student’s *t*-test or one-way analysis of variance (ANOVA) with a Tukey’s post-hoc. Differences were accepted as statistically significant at *p* < 0.05.

## References

[1] Zhao D., Yu Y., Shen Y., Liu Q., Zhao Z., Sharma R., Reiter R.J. (2019). Melatonin synthesis and function: Evolutionary history in animals and plants. Front. Endocrinol..

[2] Hardeland R., Madrid J.A., Tan D.-X., Reiter R.J. (2012). Melatonin, the circadian multioscillator system and health: The need for detailed analyses of peripheral melatonin signaling: Melatonin and peripheral oscillators. J. Pineal Res..

[3] Kleszczyński K., Kim T., Bilska B., Sarna M., Mokrzynski K., Stegemann A., Pyza E., Reiter R.J., Steinbrink K., Böhm M. (2019). Melatonin exerts oncostatic capacity and decreases melanogenesis in human MNT-1 melanoma cells. J. Pineal Res..

[4] Li T., Yang Z., Jiang S., Di W., Ma Z., Hu W., Chen F., Reiter R.J., Yang Y. (2018). Melatonin: Does it have utility in the treatment of haematological neoplasms?. Br. J. Pharmacol..

[5] Ma Z., Liu D., Di S., Zhang Z., Li W., Zhang J., Xu L., Guo K., Zhu Y., Li X. (2019). Histone deacetylase 9 downregulation decreases tumor growth and promotes apoptosis in non-small cell lung cancer after melatonin treatment. J. Pineal Res..

[6] Cabrera J., Negrín G., Estévez F., Loro J., Reiter R.J., Quintana J. (2010). Melatonin decreases cell proliferation and induces melanogenesis in human melanoma SK-MEL-1 cells. J. Pineal Res..

[7] Slominski A., Tobin D.J., Shibahara S., Wortsman J. (2004). Melanin pigmentation in mammalian skin and its hormonal regulation. Physiol. Rev..

[8] Nguyen N.T., Fisher D.E. (2019). MITF and UV responses in skin: From pigmentation to addiction. Pigment. Cell Melanoma Res..

[9] Bellei B., Maresca V., Flori E., Pitisci A., Larue L., Picardo M. (2010). p38 regulates pigmentation via proteasomal degradation of tyrosinase. J. Biol. Chem..

[10] Bae-Harboe Y.-S.C., Park H.-Y. (2012). Tyrosinase: A central regulatory protein for cutaneous pigmentation. J. Investig. Dermatol..

[11] Widlund H.R., Fisher D.E. (2003). Microphthalamia-associated transcription factor: A critical regulator of pigment cell development and survival. Oncogene.

[12] Goding C.R., Arnheiter H. (2019). MITF—The first 25 years. Genes Dev..

[13] Hartman M.L., Czyz M. (2015). MITF in melanoma: Mechanisms behind its expression and activity. Cell. Mol. Life Sci..

[14] Wang C., Zhao L., Su Q., Fan X., Wang Y., Gao S., Wang H., Chen H., Chan C.B., Liu Z. (2016). Phosphorylation of MITF by AKT affects its downstream targets and causes TP53-dependent cell senescence. Int. J. Biochem. Cell Biol..

[15] Gutknecht M., Geiger J., Joas S., Dörfel D., Salih H.R., Müller M.R., Grünebach F., Rittig S.M. (2015). The transcription factor MITF is a critical regulator of GPNMB expression in dendritic cells. Cell Commun. Signal..

[16] Hartman M.L., Czyz M. (2015). Pro-survival role of MITF in melanoma. J. Investig. Dermatol..

[17] Madsen R.R., Vanhaesebroeck B. (2020). Cracking the context-specific PI3K signaling code. Sci. Signal..

[18] Revathidevi S., Munirajan A.K. (2019). Akt in cancer: Mediator and more. Semin. Cancer Biol..

[19] Reiter R.J., Tan D.X. (2003). What constitutes a physiological concentration of melatonin?. J. Pineal Res..

[20] Liu G.-S., Peshavariya H., Higuchi M., Brewer A.C., Chang C.W.T., Chan E.C., Dusting G.J. (2012). Microphthalmia-associated transcription factor modulates expression of NADPH oxidase type 4: A negative regulator of melanogenesis. Free Radic. Biol. Med..

[21] Czarnecka A.M., Bartnik E., Fiedorowicz M., Rutkowski P. (2020). Targeted therapy in melanoma and mechanisms of resistance. Int. J. Mol. Sci..

[22] Liu R., Zhang T., Zhu G., Xing M. (2018). Regulation of mutant TERT by BRAF V600E/MAP kinase pathway through FOS/GABP in human cancer. Nat. Commun..

[23] Martin M.J., Hayward R., Viros A., Marais R. (2012). Metformin accelerates the growth of BRAF V600E-driven melanoma by upregulating VEGF-A. Cancer Discov..

[24] Mikami M., Sonoki T., Ito M., Funasaka Y., Suzuki T., Katagata Y. (2013). Glycosylation of tyrosinase is a determinant of melanin production in cultured melanoma cells. Mol. Med. Rep..

[25] Cheli Y., Ohanna M., Ballotti R., Bertolotto C. (2010). Fifteen-year quest for microphthalmia-associated transcription factor target genes. Pigment. Cell Melanoma Res..

[26] Khaled M., Larribere L., Bille K., Aberdam E., Ortonne J.-P., Ballotti R., Bertolotto C. (2002). Glycogen synthase kinase 3beta is activated by cAMP and plays an active role in the regulation of melanogenesis. J. Biol. Chem..

[27] Beurel E., Grieco S.F., Jope R.S. (2015). Glycogen synthase kinase-3 (GSK3): Regulation, actions, and diseases. Pharmacol. Ther..

[28] Zhang H.-M., Zhang Y. (2014). Melatonin: A well-documented antioxidant with conditional pro-oxidant actions. J. Pineal Res..

[29] Pelle E., Mammone T., Maes D., Frenkel K. (2005). Keratinocytes act as a source of reactive oxygen species by transferring hydrogen peroxide to melanocytes. J. Investig. Dermatol..

[30] Hu S., Huang J., Pei S., Ouyang Y., Ding Y., Jiang L., Lu J., Kang L., Huang L., Xiang H. (2019). Ganoderma lucidum polysaccharide inhibits UVB-induced melanogenesis by antagonizing cAMP/PKA and ROS/MAPK signaling pathways. J. Cell. Physiol..

[31] Sánchez-Sánchez A.M., Martín V., García-Santos G., Rodríguez-Blanco J., Casado-Zapico S., Suarez-Garnacho S., Antolín I., Rodriguez C. (2011). Intracellular redox state as determinant for melatonin antiproliferative vs cytotoxic effects in cancer cells. Free Radic. Res..

[32] Prieto-Domínguez N., Ordóñez R., Fernández A., Méndez-Blanco C., Baulies A., Garcia-Ruiz C., Fernández-Checa J.C., Mauriz J.L., González-Gallego J. (2016). Melatonin-induced increase in sensitivity of human hepatocellular carcinoma cells to sorafenib is associated with reactive oxygen species production and mitophagy. J. Pineal Res..

[33] Shen Y.-Q., Guerra-Librero A., Fernandez-Gil B.I., Florido J., García-López S., Martinez-Ruiz L., Mendivil-Perez M., Soto-Mercado V., Acuña-Castroviejo D., Ortega-Arellano H. (2018). Combination of melatonin and rapamycin for head and neck cancer therapy: Suppression of AKT/mTOR pathway activation, and activation of mitophagy and apoptosis via mitochondrial function regulation. J. Pineal Res..

[34] Jain A.K., Jaiswal A.K. (2007). GSK-3beta acts upstream of Fyn kinase in regulation of nuclear export and degradation of NF-E2 related factor 2. J. Biol. Chem..

[35] Cuadrado A. (2015). Structural and functional characterization of Nrf2 degradation by glycogen synthase kinase 3/β-TrCP. Free Radic. Biol. Med..

[36] Alvarez-Artime A., Cernuda-Cernuda R., Artime-Naveda F., Cepas V., Gonzalez-Menendez P., Fernadez-Vega S., Quiros-Gonzalez I., Sainz R.M., Mayo J.C. (2020). Melatonin-induced cytoskeleton reorganization leads to inhibition of melanoma cancer cell proliferation. Int. J. Mol. Sci..

[37] Slominski A., Pruski D. (1993). Melatonin inhibits proliferation and melanogenesis in rodent melanoma cells. Exp. Cell Res..

[38] Kim T.-K., Lin Z., Tidwell W.J., Li W., Slominski A.T. (2015). Melatonin and its metabolites accumulate in the human epidermis in vivo and inhibit proliferation and tyrosinase activity in epidermal melanocytes in vitro. Mol. Cell. Endocrinol..

[39] Souza A.V., Visconti M.A., de Lauro Castrucci A.M. (2003). Melatonin biological activity and binding sites in human melanoma cells. J. Pineal Res..

[40] Kim T.K., Kleszczynski K., Janjetovic Z., Sweatman T., Lin Z., Li W., Reiter R.J., Fischer T.W., Slominski A.T. (2013). Metabolism of melatonin and biological activity of intermediates of melatoninergic pathway in human skin cells. FASEB J..

[41] Negroiu G., Branza-Nichita N., Petrescu A.J., Dwek R.A., Petrescu S.M. (1999). Protein specific N-glycosylation of tyrosinase and tyrosinase-related protein-1 in B16 mouse melanoma cells. Biochem. J..

[42] Takeda K., Takemoto C., Kobayashi I., Watanabe A., Nobukuni Y., Fisher D.E., Tachibana M. (2000). Ser298 of MITF, a mutation site in Waardenburg syndrome type 2, is a phosphorylation site with functional significance. Hum. Mol. Genet..

[43] Klein P.S., Melton D.A. (1996). A molecular mechanism for the effect of lithium on development. Proc. Natl. Acad. Sci. USA.

[44] Sklirou A.D., Gaboriaud-Kolar N., Papassideri I., Skaltsounis A.-L., Trougakos I.P. (2017). 6-bromo-indirubin-3′-oxime (6BIO), a Glycogen synthase kinase-3β inhibitor, activates cytoprotective cellular modules and suppresses cellular senescence-mediated biomolecular damage in human fibroblasts. Sci. Rep..

[45] Niu C., Yin L., Aisa H. (2018). Novel furocoumarin derivatives stimulate melanogenesis in B16 melanoma cells by up-regulation of MITF and TYR family via Akt/GSK3β/β-catenin signaling pathways. Int. J. Mol. Sci..

[46] Cross D.A., Alessi D.R., Cohen P., Andjelkovich M., Hemmings B.A. (1995). Inhibition of glycogen synthase kinase-3 by insulin mediated by protein kinase B. Nature.

[47] Khaled M., Larribere L., Bille K., Ortonne J.P., Ballotti R., Bertolotto C. (2003). Microphthalmia associated transcription factor is a target of the phosphatidylinositol-3-kinase pathway. J. Investig. Dermatol..

[48] Lu Y.-X., Chen D.-L., Wang D.-S., Chen L.-Z., Mo H.-Y., Sheng H., Bai L., Wu Q.-N., Yu H.-E., Xie D. (2016). Melatonin enhances sensitivity to fluorouracil in oesophageal squamous cell carcinoma through inhibition of Erk and Akt pathway. Cell Death Dis..

[49] McCubrey J.A., Steelman L.S., Bertrand F.E., Davis N.M., Sokolosky M., Abrams S.L., Montalto G., D’Assoro A.B., Libra M., Nicoletti F. (2014). GSK-3 as potential target for therapeutic intervention in cancer. Oncotarget.

[50] Galano A., Tan D.X., Reiter R.J. (2013). On the free radical scavenging activities of melatonin’s metabolites, AFMK and AMK. J. Pineal Res..

[51] Fernandez-Gil B.I., Guerra-Librero A., Shen Y.-Q., Florido J., Martínez-Ruiz L., García-López S., Adan C., Rodríguez-Santana C., Acuña-Castroviejo D., Quiñones-Hinojosa A. (2019). Melatonin enhances cisplatin and radiation cytotoxicity in head and neck squamous cell carcinoma by stimulating mitochondrial ROS generation, apoptosis, and autophagy. Oxid. Med. Cell. Longev..

[52] Kim H.-Y., Sah S.K., Choi S.S., Kim T.-Y. (2018). Inhibitory effects of extracellular superoxide dismutase on ultraviolet B-induced melanogenesis in murine skin and melanocytes. Life Sci..

[53] Kim H.-E., Lee S.-G. (2013). Induction of ATP synthase β by H_2_O_2_ induces melanogenesis by activating PAH and cAMP/CREB/MITF signaling in melanoma cells. Int. J. Biochem. Cell Biol..

[54] Cho H., Kim O., Lee Y., Kang L.-J., Nguyen C.N., Ishihara A., Kim H.-E. (2017). Feruloylserotonin inhibits hydrogen peroxide-induced melanogenesis and apoptosis in B16F10 and SK-Mel-2 melanoma cells. Biochem. Biophys. Res. Commun..

[55] Liu F., Fu Y., Meyskens F.L. (2009). MiTF regulates cellular response to reactive oxygen species through transcriptional regulation of APE-1/Ref-1. J. Investig. Dermatol..

[56] Vriend J., Reiter R.J. (2015). The Keap1-Nrf2-antioxidant response element pathway: A review of its regulation by melatonin and the proteasome. Mol. Cell. Endocrinol..

[57] Janjetovic Z., Nahmias Z.P., Hanna S., Jarrett S.G., Kim T.-K., Reiter R.J., Slominski A.T. (2014). Melatonin and its metabolites ameliorate ultraviolet B-induced damage in human epidermal keratinocytes. J. Pineal Res..

[58] Janjetovic Z., Jarrett S.G., Lee E.F., Duprey C., Reiter R.J., Slominski A.T. (2017). Melatonin and its metabolites protect human melanocytes against UVB-induced damage: Involvement of NRF2-mediated pathways. Sci. Rep..

[59] Shin J.-M., Kim M.Y., Sohn K.-C., Jung S.-Y., Lee H.-E., Lim J.W., Kim S., Lee Y.-H., Im M., Seo Y.-J. (2014). Nrf2 negatively regulates melanogenesis by modulating PI3K/Akt signaling. PLoS ONE.

[60] Zhao Z., Lu C., Li T., Wang W., Ye W., Zeng R., Ni L., Lai Z., Wang X., Liu C. (2018). The protective effect of melatonin on brain ischemia and reperfusion in rats and humans: In vivo assessment and a randomized controlled trial. J. Pineal Res..

[61] Pan H., Wang H., Jia Y., Wang Q., Li L., Wu Q., Chen L. (2017). VPA and MEL induce apoptosis by inhibiting the Nrf2-ARE signaling pathway in TMZ-resistant U251 cells. Mol. Med. Rep..

[62] Ma Y., Wang X., Chen J., Li B., Hur E.-M., Saijilafu (2017). Differential roles of glycogen synthase kinase 3 subtypes alpha and beta in cortical development. Front. Mol. Neurosci..

[63] Thangasamy T., Sittadjody S., Limesand K.H., Burd R. (2008). Tyrosinase overexpression promotes ATM-dependent p53 phosphorylation by quercetin and sensitizes melanoma cells to dacarbazine. Anal. Cell. Pathol..

[64] Wang Y.M., Jin B.Z., Ai F., Duan C.H., Lu Y.Z., Dong T.F., Fu Q.L. (2012). The efficacy and safety of melatonin in concurrent chemotherapy or radiotherapy for solid tumors: A meta-analysis of randomized controlled trials. Cancer Chemother. Pharmacol..

